# Exacerbation of ichthyosis vulgaris phenotype by co-inheritance of *STS* and *FLG* mutations in a Chinese family with ichthyosis: a case report

**DOI:** 10.1186/s12881-018-0642-5

**Published:** 2018-07-18

**Authors:** Xiong Wang, Lu Tan, Na Shen, Yanjun Lu, Ying Zhang

**Affiliations:** 1 Department of Laboratory Medicine, Tongji Hospital, Tongji Medical College, Huazhong University of Science and Technology, Wuhan, 430030 China; 2 Key Laboratory for Molecular Diagnosis of Hubei Province, The Central Hospital of Wuhan, Tongji Medical College, Huazhong University of Science and Technology, Wuhan, 430014 Hubei China; 3 Department of Rehabilitation, Zhongnan Hospital of Wuhan University, Wuhan, 430000 China

**Keywords:** STS, FLG, Ichthyosis, Mutation, Co-inheritance

## Abstract

**Background:**

X-linked ichthyosis (XLI) is a recessive keratinization condition caused by deficient activity of steroid-sulfatase due to mutations in steroid sulfatase (*STS*) gene located on the X chromosome. In contrast, ichthyosis vulgaris (IV) is caused by filaggrin deficiency due to semi-dominant loss-of-function mutations of filaggrin (*FLG*) gene. Filaggrin defects could synergize with XLI to exacerbate its phenotype.

**Case presentation:**

We report a Chinese family with patients presenting diverse phenotype of Keratosis pilaris. A next-generation sequencing panel interrogating 25 ichthyosis related genes with sequencing coverage of the coding regions and splice site junctions, was applied to screen genetic mutations. A gross deletion encompassing the *STS* gene ranging from exon 1–10 and the *FLG* c.3321delA mutation were identified in a 31-year old male proband, one of his sister, and his mother, and all the three patients showed obvious symptom. The deletion of *STS* gene was confirmed by real-time quantitative PCR. The proband’s another sister and his two nephews carried only *FLG* c.3321delA mutation. Patients carried both mutations presented more severe symptom, while those only carried *FLG* c.3321delA mutation showed slight or normal phenotype.

**Conclusions:**

In conclusion, we found that the IV phenotype was exacerbated by co-inheritance of *STS* and *FLG* mutations in a Chinese family with ichthyosis. Other genomic regions no included in the study might be also involved in phenotypic modifications.

## Background

Ichthyosis vulgaris (IV, OMIM #146700) is the most frequent genetic disorder of ichthyosis caused by filaggrin deficiency due to semi-dominant loss-of-function mutations of filaggrin (*FLG*) gene, which affects around 1 in 250 people [1]. IV is characterized by palmar and plantar hyperlinearity, keratosis pilaris, hyperkeratosis, xerosis, and excess scaling. The phenotype is most pronounced in winter or dry climates [2]. The onset of IV could occur at an early age, and become apparent between 3 months and 5 years of age in patients with positive family history [3]. IV is inherited as a semi-dominant manner with variable penetrance [4].

X-linked ichthyosis (XLI, OMIM#308100) is one of the second most prevalent type of ichthyosis caused by steroid sulfatase (STS) deficiency due to mutation of *STS* gene located on the X chromosome, affecting approximately 1:2000 to 1:6000 males worldwide. Female carriers with few exceptions do not manifest XLI [5]. XLI is clinically characterized by general scaling of the skin in which the scalp, ears, neck, trunk, and limbs are affected. It consists of mild-to-moderate polygonal, dark brown, adherent and regular scales, which is prominent on the lateral aspects of the trunk and the lower limbs. Extracutaneous signs such as corneal opacities, cryptorchidism, attention deficit hyperactivity, male baldness pattern are frequent, especially corneal opacities and cryptorchidism [6].

Clinically it may be difficult to distinguish XLI from IV [4]. Moreover, as a modifying factors, *FLG* mutations could exacerbate XLI phenotype, and increased prevalence of filaggrin deficiency has been observed in XLI patients [7–9]. Recently, next-generation sequencing (NGS) has been widely applied as a rapid genetic diagnosis to identify novel mutations in patients with ichthyosis [10, 11].

In the current study, we described a male proband affected XLI and IV, and female IV patients carrying heterozygous *STS* mutation simultaneously. Moreover, both male and female patients suffered IV were found in this family. The male proband presented the most severe ichthyosis phenotype. Female IV patients carrying heterozygous *STS* mutation showed more severe phenotype than IV patients without *STS* mutation. This study confirmed the synergic effect between XLI and IV, and female XLI carriers could also exacerbate their IV phenotype.

## Case presentation

### Patients

**Fig. 1:**
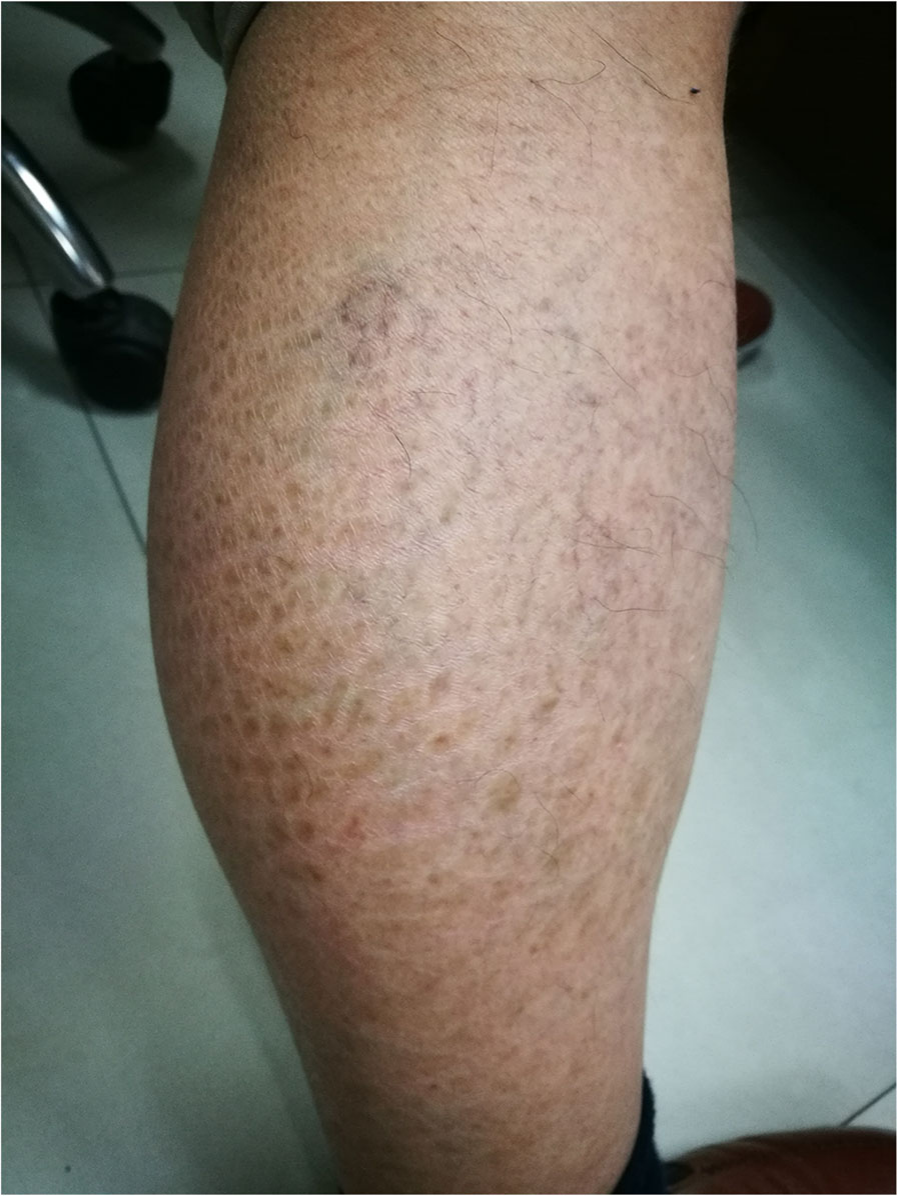
Clinical appearance of the proband co-inherited with XLI and IV. The proband had obvious, widespread and hyperpigmented ichthyosis in extensor surfaces of lower limbs even after treatment

**Fig. 2:**
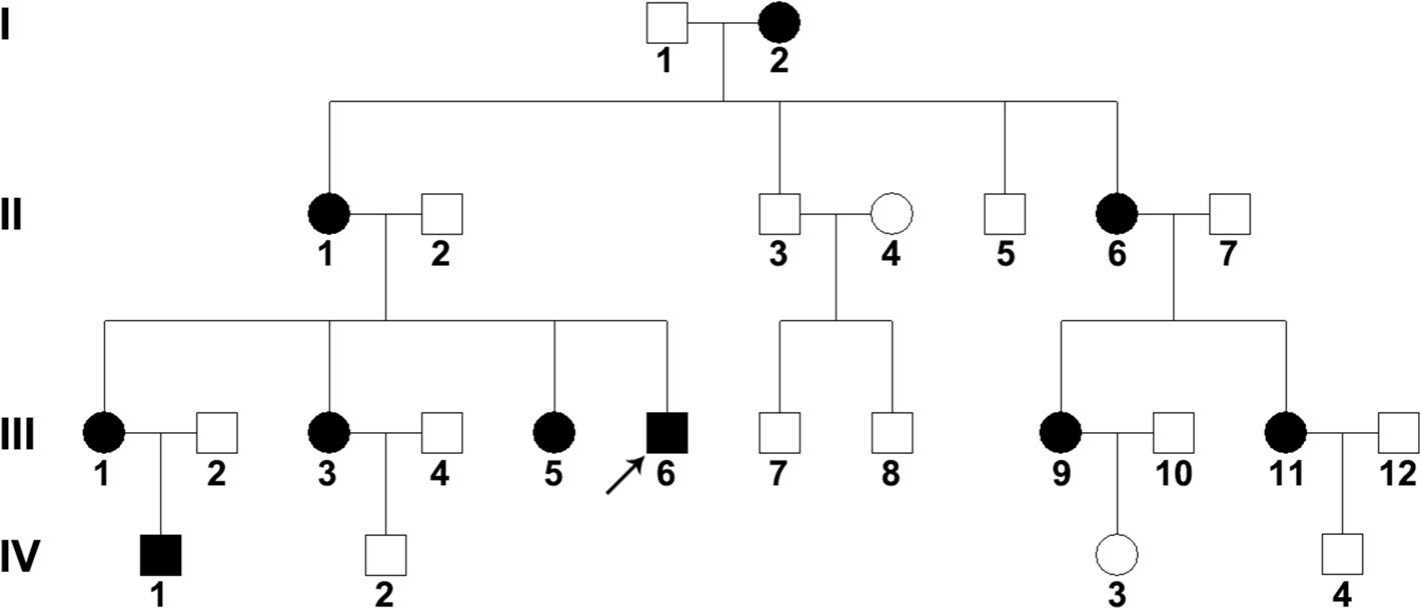
The Chinese XLI/IV coexisting pedigree. Squares represent men, circles represent women, and arrowheads indicate the proband. Symbols in dark represent patients. I, II, III, IV represent four generations

The 31-year old male proband presented with symmetrical scaling when he was young, which was more prominent on the extensor surfaces of the limbs, along with dark brown, tightly adherent polygonal scales (Fig. 1). The soles and palms were unaffected. The proband is the fourth child, and his mother had a similar but less severe phenotype. His father was unaffected. Two of the elder sister had similar phenotype with their mother, and one of them had a 4-year old boy without phenotype. Another elder sister presented slight scaling, whose 12-year old boy presented slight phenotype. In the extended family, 4 affected females had a slight phenotype. The family tree was drawn (Fig. 2).

**Table 1:** *STS* and *FLG* mutation in a Chinese family with ichthyosis

No.	Relation	Gender	Age	Symptom	FLG c.3321delA	STS Exon 1–10 Del
II-1	Mother	Female	70	Obvious	Heterozygous	Heterozygous
II-2	Father	Male	69	Normal	Wild type	Wild type
III-1	First sister	Female	40	Slight	Heterozygous	Wild type
III-3	Second sister	Female	37	Obvious	Heterozygous	Heterozygous
III-5	Third sister	Female	33	Obvious	Not available	Not available
III-6	Proband	Male	31	Obvious	Heterozygous	Hemizygous
IV-1	Son of III-1	Male	12	Slight	Heterozygous	Wild type
IV-4	Son of III-3	Male	4	Normal	Heterozygous	Wild type

### Mutation analysis

To investigate the genetic defects for patients with ichthyosis, a panel of 25 genes (*ABCA12*, *ALOX12B*, *ALOXE3*, *CLDN1*, *COL17A1*, *COL7A1*, *CYP4F22*, *FLG*, *ITGA6*, *ITGB4*, *KRT14*, *KRT5*, *LAMA3*, *LAMB3*, *LAMC2*, *MBTPS2*, *NIPAL4*, *PLEC*, *PNPLA1*, *SLC27A4*, *SNAP29*, *SPINK5*, *ST14*, *STS*, and *TGM1*) underlying the most common genetic defects for ichthyosis was detected by NGS (BGI-Wuhan). Briefly, all exons with the adjacent 10 bp introns of the 25 genes covering 100,596 bp in length listed above were captured by using a microarray chip, and were further sequenced with Illumina HiSeq2000. Base calling was performed with the Illumina Pipeline. Mutations were identified using the BWA (Burrows Wheeler Aligner) software package against the hg19 human genome reference. The average sequencing depth for target region was 272.2 -fold, and the average coverage was 98.84%. 97.02% of the target region was sequenced for more than 30-fold. Mutation identified by NGS was validated by Sanger sequencing. The detection of exonic deletions using target capture and deep sequencing data was performed using the script for the detection of exonic deletions as previously described [12]. Deletion of *STS* gene was further validated by real-time quantitative PCR of genomic DNA isolated from peripheral blood.

### *STS* deletion of exon 1–10 and *FLG* c.3321delA mutation

A total of 153 mutations were identified by NGS in the proband. After data processing and filtering referred to inherited model, minor allele frequency (MAF) in 1000G, ExAC, and gnomAD databases, splice effect, computer prediction and so on, hemizygous *STS* deletion of exon 1–10 (NM_000351) and heterozygous *FLG* NM_002016: c.3321delA (p.Ser1107SerfsTer15) frameshift mutation were identified as pathogenic mutations in the proband. The deletion of *STS* gene was confirmed by real-time quantitative PCR using a healthy female and a male subject as control. *FLG* c.3321delA mutation was confirmed by Sanger sequencing.

**Fig. 3:**
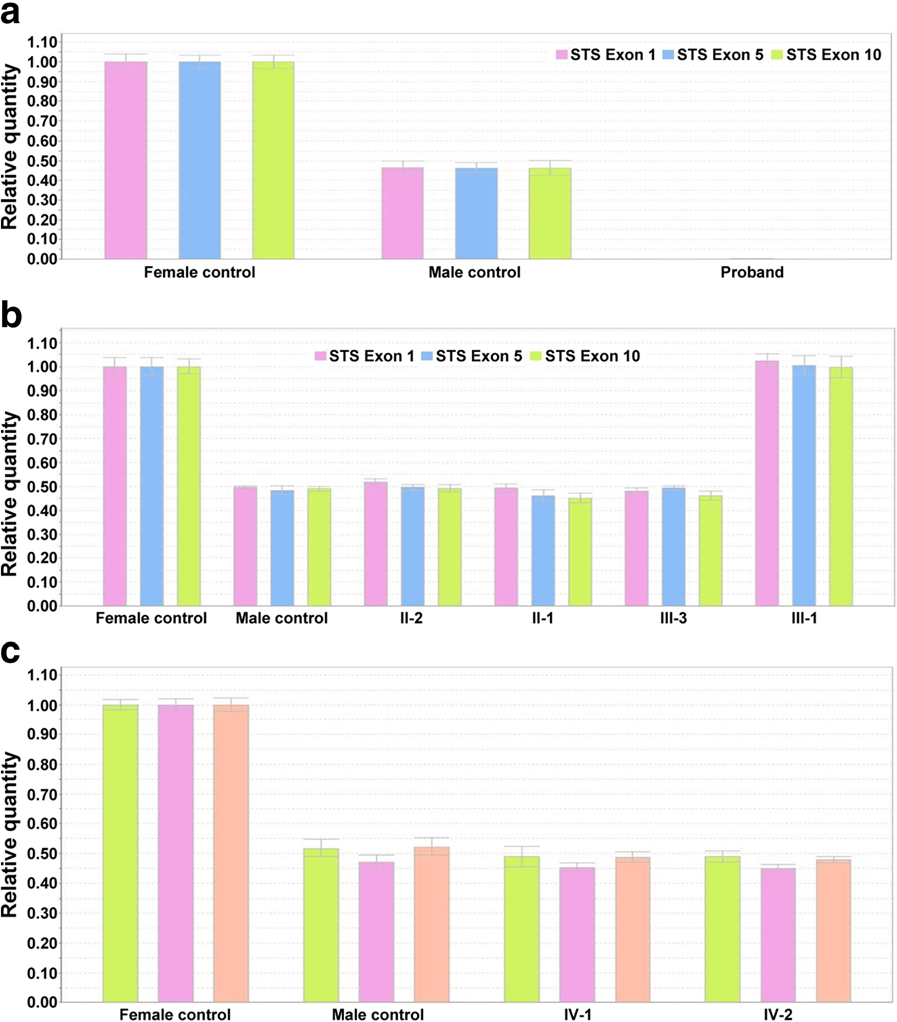
Expression of *STS* gene in a Chinese family with ichthyosis. The expression of *STS* was detected by real-time quantitative PCR. Primers were designed to amplify exon 1, 5, and 10 of *STS* gene. **a** Proband, **b** Proband’s parents and sisters, **c** Proband’s nephew

**Fig. 4:**
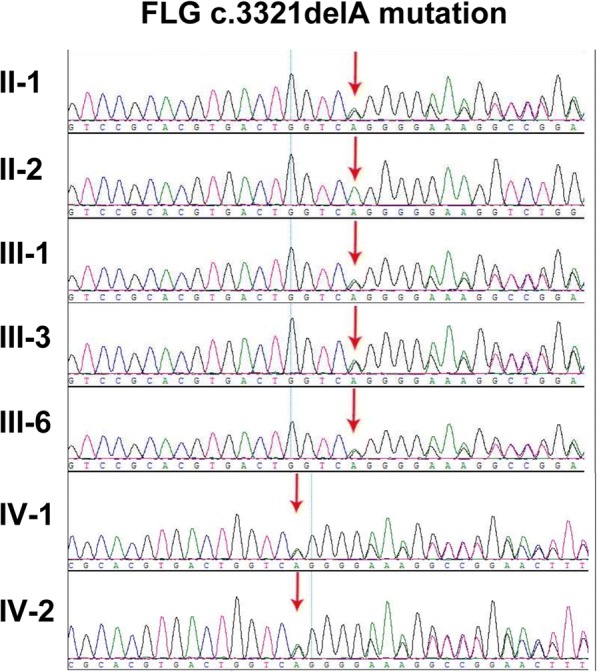
*FLG* c.3321delA mutation in a Chinese family with ichthyosis. Sanger sequencing was performed for this family members

Several family members were included. *STS* deletion of exon 1–10 and *FLG* c.3321delA mutation were validated in these included family members (Figs 3 and 4).

### Genotype and phenotype correlation

In this family, the male proband presented the most severe scaling (Table 1). He carried hemizygous *STS* deletion of exon 1–10 and heterozygous *FLG* c.3321delA mutation. His mother and one elder sister showed obvious but less sever symptom than the proband harbored heterozygous *STS* deletion of exon 1–10 and heterozygous *FLG* c.3321delA mutation. Another elder sister and 12-year old nephew showed slight phenotype carried only heterozygous *FLG* c.3321delA mutation. Another 4-year old nephew carried only heterozygous *FLG* c.3321delA mutation had no clinical symptom yet. The proband’s father was unaffected and neither mutation was detected.

## Discussion and conclusions

STS deficiency results in accumulation of cholesterol sulfate in the outer layers of the skin, inducing intercellular cohesion and scaling in XLI [13]. Up to 90% of the XLI patients presented complete deletion of the entire *STS* gene, and deletions could even extend to neighboring genes sometimes, leading to continuous gene syndromes [6, 14]. Less frequent point mutations have been reported as well [15, 16]. In this study, *STS* deletion of exon 1–10 has been identified by NGS, and further confirmed by real-time PCR via amplifying exon 1, 5, 10 in genomic DNA, which were widely used to detect *STS* deletion of exon 1–10 [6, 17]. The proband was hemizygote, one of his elder sister and their mother were heterozygotes. They all showed entire deletion of the *STS* gene, consistent with previous studies.

Genetic linkage analyses on IV patients mapped the *FLG* gene to the epidermal differentiation complex on chromosome 1q21 [18]. Loss-of-function mutations of the *FLG* gene have been identified to underlie IV, which is inherited in a semi-dominant model with 83–96% penetrance where heterozygotes have mild sub-clinical phenotype compared with homozygotes who with more prominent ichthyosis [1]. *FLG* mutations tend to be population specific. S2554X and 3321delA mutations were prevalent mutations in Asians, including Japanese and Chinese IV patients [19]. In this study, heterozygous *FLG* 3321delA mutation was observed in several patients. Some patients only carried heterozygous *FLG* 3321delA mutation, and some patients harbored both *FLG* 3321delA mutation and *STS* deletion.

Accumulating evidence indicates that *FLG* mutations may act as modifying factors of *STS* mutation, which could exacerbate XLI phenotype. Süßmuth K et al. found that the prevalence of *FLG* mutations was significantly increased in XLI patients compared to a population-based control cohort [8]. Liao H et al. suggested that different pathways disrupting epidermal differentiation may increase phenotypic severity [20]. Zhang Q et al. reported that filaggrin defects may synergize with deficiency of STS to exacerbate the XLI phenotype [9]. Ramesh R et al. also demonstrated the modifying function of *FLG* null alleles on XLI [7].

In this family, the male patients carried both heterozygous *FLG* 3321delA mutation and hemizygous STS deletion was the most severe affected, indicating the synergic effect of *FLG* and *STS* mutation.

Moreover, among the female patients, those carried two mutations were more severe affected than those harboring only heterozygous *FLG* 3321delA mutation, although previous studies reported that female carriers of STS mutation were seldom affected. The second elder sister who was 37-year old and carried both mutations presented more sever scaling than the first elder sister who was 40-year old and only harbored *FLG* mutation. These results suggest that female XLI carriers could also exacerbate their IV phenotype, and the effect of age was excluded.

In conclusion, we found that the IV phenotype was exacerbated by co-inheritance of *STS* and *FLG* mutations in a Chinese family with ichthyosis. Other genomic regions no included in the study might be also involved in phenotypic modifications.

## Abbreviations

1000G: 1000 Genomes Project
BWA: Burrows Wheeler Aligner
ExAC: The Exome Aggregation Consortium
FLG: filaggrin
gnomAD: The Genome Aggregation Database
IV: Ichthyosis vulgaris
MAF: Minor allele frequency
NGS: Next-generation sequencing
STS: Steroid sulfatase
XLI: X-linked ichthyosis
